# (η^5^-Penta­methyl­cyclo­penta­dien­yl)(η^6^-*p*-toluene­sulfonamide)ruthenium(II) tetra­phenyl­borate

**DOI:** 10.1107/S1600536808037689

**Published:** 2008-11-20

**Authors:** Bradley T. Loughrey, Michael L. Williams, Sally-Ann Poulsen, Peter C. Healy

**Affiliations:** a Eskitis Institute for Cell and Molecular Therapies, Griffith University, Brisbane 4111, Australia

## Abstract

The crystal structure of the title compound, [Ru(C_10_H_15_)(C_7_H_9_NO_2_S)]C_24_H_20_B, has been determined as part of our investigation into the structural and biological properties of organometallic Ru^II^–arene–Cp* complex salts of the type [*R*-PhRuCp*]^+^·*X* ^−^ (where Cp* is penta­methyl­cyclo­penta­diene). Tethering the RuCp* group to the benzene ring of *p*-toluene­sulfonamide results in only minor changes to the mol­ecular geometry of the sulfonamide, but, together with crystallization as the [BPh_4_]^−^ salt, effectively blocks involvement of the sulfonamide group in N—H⋯O hydrogen-bonding networks.

## Related literature

For related literature, see: Navarro Clemente *et al.* (2002 ▶); Ferguson & Glidewell (1988 ▶); Gemel *et al.* (1996 ▶); Loughrey *et al.* (2008 ▶); Moreno *et al.* (2008 ▶); Salmon *et al.* (2007 ▶); Zerbe *et al.* (2005 ▶).
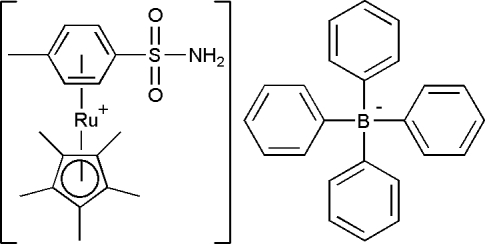


## Experimental

### 

#### Crystal data

[Ru(C_10_H_15_)(C_7_H_9_NO_2_S)]C_24_H_20_B
*M* *_r_* = 726.72
Monoclinic, 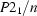

*a* = 12.2254 (2) Å
*b* = 13.4685 (2) Å
*c* = 22.9204 (5) Å
β = 104.000 (2)°
*V* = 3661.92 (12) Å^3^
*Z* = 4
Mo *K*α radiation
μ = 0.52 mm^−1^
*T* = 296 K
0.43 × 0.30 × 0.23 mm

#### Data collection

Oxford-Diffraction GEMINI S ultra diffractometer
Absorption correction: multi-scan (*CrysAlis RED*; Oxford Diffraction, 2007 ▶) *T*_min_ = 0.807, *T*_max_ = 0.890
17618 measured reflections
8151 independent reflections
6209 reflections with *I* > 2σ(*I*)
*R*_int_ = 0.024

#### Refinement

*R*[*F* ^2^ > 2σ(*F* ^2^)] = 0.037
*wR*(*F* ^2^) = 0.089
*S* = 1.04
8151 reflections
430 parameters
H-atom parameters constrained
Δρ_max_ = 0.51 e Å^−3^
Δρ_min_ = −0.29 e Å^−3^

### 

Data collection: *CrysAlis CCD* (Oxford Diffraction, 2007 ▶); cell refinement: *CrysAlis RED* (Oxford Diffraction, 2007 ▶); data reduction: *CrysAlis RED*; program(s) used to solve structure: *SIR97* (Altomare *et al.*, 1999 ▶); program(s) used to refine structure: *SHELXL97* (Sheldrick, 2008 ▶); molecular graphics: *ORTEP-3 for Windows* (Farrugia, 1997 ▶); software used to prepare material for publication: *PLATON* (Spek, 2003 ▶).

## Supplementary Material

Crystal structure: contains datablocks global, I. DOI: 10.1107/S1600536808037689/bh2206sup1.cif

Structure factors: contains datablocks I. DOI: 10.1107/S1600536808037689/bh2206Isup2.hkl

Additional supplementary materials:  crystallographic information; 3D view; checkCIF report

## Figures and Tables

**Fig. 1.**
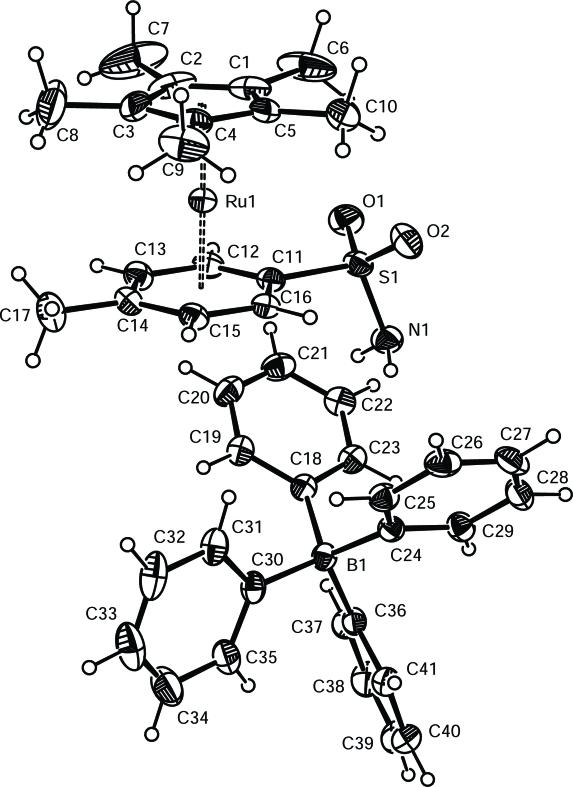
The structure of (I), with atom labels and 30% probability displacement ellipsoids for the non-H atoms.

**Fig. 2.**
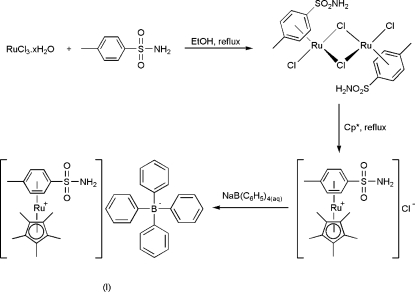
Synthetic route for (I).

## supplementary crystallographic information

### Comment

As part of our investigation into the synthesis, structural characterization and biological properties of ionic Ru(II) organometallic complexes [*R*-PhRuCp*]*X* (Loughrey *et al.*, 2008), we have prepared and characterized the tetraphenylborate salt of the organometallic sandwich compound of RuCp* and *p*-toluenesulfonamide, (I), and determined its crystal structure (Fig. 1). While covalent bonding of ruthenocene to sulfonamides has been reported previously (Salmon *et al.*, 2007), this is the first recorded synthesis and structure determination of a ruthenium based organometallic sulfonamide where the metal has been tethered directly to an aromatic sulfonamide pharmacaphore.

The compound was prepared in 75% yield through the reaction of RuCl_3_.xH_2_O with an excess of the aromatic sulfonamide ligand in ethanol. This prompts formation of the labile chlorido-bridged dimer which can undertake ligand exchange of the µ-Cl ligands with a Cp* ligand and formation of the cation which can be readily isolated as the tetraphenylborate salt through addition of an aqueous solution of sodium tetraphenylborate (Fig. 2).

The structural parameters of the cation in (I) are similar to those reported for other [(arene)RuCp*]^+^ complexes (Gemel *et al.*, 1996; Navarro Clemente *et al.*, 2002; Loughrey *et al.*, 2008) with Ru—C distances of 2.177 (4)–2.196 (3) Å (Cp*) and 2.193 (3)–2.240 (3) Å (Ph). The average value of 1.41 (1) Å for the C—C bond lengths in the benzene ring is 0.02Å longer than the average of 1.39 (1) Å observed for the parent sulfonamide (Zerbe *et al.*, 2005), while the bond lengths in the SO_2_NH_2_ groups do not differ significantly. As illustrated in Fig. 1, the S═O bonds are directed towards, and the NH_2_ group away, from the Cp* ring with torsion angles O1—S1—C11—C12 16.0 (2)°, O2—S1—C11—C16 -31.7 (2)° and N1—S1—C11—C16 82.9 (2)°. A feature of the present structure is the complete blocking, by the Cp* group and the [BPh_4_]^-^ anion, of the N—H···O hydrogen bonding networks that are characteristic of other structural studies of the sulfonamide (Zerbe *et al.*, 2005; Ferguson & Glidewell, 1988).

Comparison of the ^1^H NMR spectra for (I) and its parent sulfonamide shows the aromatic protons to experience an upfield shift of *ca* 1.5 p.p.m. which can be attributed to the presence of the diamagnetic Ru(II) shielding the nuclei of the aromatic protons. The sulfonamide protons experience a downfield shift of 0.49 p.p.m. which is also attributable to the loss of electron density from the sulfonamide group due to π-donor backbonding of the ligand with the *d*-orbitals of the metal (Moreno *et al.*, 2008).

### Experimental

4-Methylbenzenesulfonamide (1.04 g, 6.08 mmol) was added to a solution of ruthenium trichloride hydrate (0.20 g) in ethanol (20 ml) under argon. The resulting solution was refluxed for a period of 10 h to give a dark blue solution. Pentamethylcyclopentadiene (Cp*, 0.3 ml, 1.88 mmol) was added to solution and the mixture refluxed for a further 10 h. The solvent was concentrated *in vacuo* with the remaining residue being redissolved in an ether/water partition (20 ml/20 ml). The aqueous portion was retained and washed with a further three aliquots of diethyl ether (20 ml). The aqueous layer was then mixed slowly with an aqueous solution of sodium tetraphenylborate (5 ml, 0.30 *M*). The resulting precipitate was filtered from solution and redissolved in a minimum volume of acetone. This solution was filtered through a short alumina column (neutral, 150 mesh) using acetone as the eluent. The solution was concentrated *in vacuo* and the product was recrystallized through addition of a minimum quantity of cold water. The resulting crystalline precipitate was then filtered from solution and dried *in vacuo*. Yield = 0.409 g, 75%. Crystals suitable for X-ray diffraction studies were grown from a saturated solution of methanol following slow diffusion with diethyl ether. *M*.p. 245°C (decomp). Analysis: Calcd for C_41_H_44_O_2_NSBRu: C 67.8, H 6.11, N 1.93, S 4.41%. Found C 67.4, H 5.87, N 1.92, S 4.68%.

^1^H NMR (400 MHz, *d*_6_-DMSO, 298 K), δ p.p.m. 1.88 (s, 15H, C5(C_5_H_15_)), 2.19 (s, 3H, CH_3_), 6.03–6.05 (m, 2H, C_6_H_4_*ortho*), 6.21–6.23 (m, 2H, C_6_H_4_*meta*), 6.75–6.80 (m, 4H, B(C_6_H_5_)_4_*para*), 6.88–6.93 (m, 8H, B(C_6_H_5_)_4_*meta*), 7.14–7.19 (m, 8H, B(C_6_H_5_)_4_*ortho*), 7.85 (s, 2H, SO_2_NH_2_). ^13^C{^1^H} NMR (100 MHz, *d*_6_-DMSO), δ p.p.m. 9.69 (C_5_(C_5_H_15_)), 17.35 (CH_3_), 84.11 (2CH, aromatic), 87.85 (2CH, aromatic), 97.14 (C_5_(C_5_H_15_)), 101.70 (C—CH_3_), 104.69 (C—SO_2_NH_2_), 121.48 (4CH, B(C_6_H_5_)_4_), 125.28 (8CH, B(C_6_H_5_)_4_), 135.50 (8CH, B(C_6_H_5_)_4_), 162.35, 163.01, 163.65, 164.31 (4CH, B(C_6_H_5_)_4_, signals split by ^11^B). ESMS (m/*z*): +ve ion, calcd *m*/*z* for [(η^5^-C_5_(CH_3_)_5_)Ru (η^6^-CH_3_C_6_H_4_SO_2_NH_2_)^+^]: 407.56, found: 407.98 (100%), -ve ion, calcd *m*/z for B(C_6_H_5_)_4_^-^: 319.25, found: 319.11 (100%).

### Refinement

H atoms attached to carbon were constrained as riding atoms, with C—H set to 0.94–96 Å. *U*_iso_(H) values were set to 1.2*U*_eq_ (aromatic) and 1.5*U*_eq_ (alkyl) of the parent atom. The N protons were located in Fourier difference maps and constrained as riding atoms with N—H set to 0.86Å and *U*_iso_(H) values set to 1.2*U*_eq_ of the parent atom.

### Crystal data

**Table:**

[Ru(C_10_H_15_)(C_7_H_9_NO_2_S)]C_24_H_20_B	*F*_000_ = 1512
*M**_r_* = 726.72	*D*_x_ = 1.318 Mg m^−^^3^
Monoclinic, *P*2_1_/*n*	Melting point: 518 K
Hall symbol: -P 2yn	Mo *K*α radiation λ = 0.71070 Å
*a* = 12.2254 (2) Å	Cell parameters from 9519 reflections
*b* = 13.4685 (2) Å	θ = 3.1–27.5º
*c* = 22.9204 (5) Å	µ = 0.52 mm^−^^1^
β = 104.000 (2)º	*T* = 296 K
*V* = 3661.92 (12) Å^3^	Block, brown
*Z* = 4	0.43 × 0.30 × 0.23 mm

### Data collection

**Table:**

Oxford-Diffraction GEMINI S Ultra diffractometer	8151 independent reflections
Radiation source: Enhance (Mo) X-ray Source	6209 reflections with *I* > 2σ(*I*)
Monochromator: graphite	*R*_int_ = 0.024
Detector resolution: 16.0774 pixels mm^-1^	θ_max_ = 27.6º
*T* = 296 K	θ_min_ = 3.1º
ω and φ scans	*h* = −15→15
Absorption correction: multi-scan(CrysAlis RED; Oxford Diffraction, 2007)	*k* = −15→17
*T*_min_ = 0.807, *T*_max_ = 0.890	*l* = −29→29
17618 measured reflections	

### Refinement

**Table:**

Refinement on *F*^2^	Secondary atom site location: difference Fourier map
Least-squares matrix: full	Hydrogen site location: inferred from neighbouring sites
*R*[*F*^2^ > 2σ(*F*^2^)] = 0.037	H-atom parameters constrained
*wR*(*F*^2^) = 0.089	*w* = 1/[σ^2^(*F*_o_^2^) + (0.0489*P*)^2^ + 0.272*P*] where *P* = (*F*_o_^2^ + 2*F*_c_)/3
*S* = 1.04	(Δ/σ)_max_ = 0.007
8151 reflections	Δρ_max_ = 0.51 e Å^−^^3^
430 parameters	Δρ_min_ = −0.29 e Å^−^^3^
Primary atom site location: structure-invariant direct methods	

### Fractional atomic coordinates and isotropic or equivalent isotropic displacement parameters (Å^2^)

**Table:**

	*x*	*y*	*z*	*U*_iso_*/*U*_eq_	
Ru1	0.38014 (1)	−0.21168 (1)	0.17469 (1)	0.0365 (1)	
S1	0.35157 (5)	0.04626 (5)	0.20161 (3)	0.0456 (2)	
O1	0.23845 (15)	0.05536 (15)	0.16834 (11)	0.0647 (7)	
O2	0.37719 (18)	0.02787 (14)	0.26438 (9)	0.0627 (7)	
N1	0.41631 (19)	0.14599 (16)	0.19196 (11)	0.0542 (8)	
C1	0.2334 (2)	−0.2331 (2)	0.21247 (17)	0.0621 (12)	
C2	0.2250 (3)	−0.2969 (3)	0.16296 (16)	0.0739 (11)	
C3	0.3198 (3)	−0.3637 (2)	0.17651 (14)	0.0624 (10)	
C4	0.3851 (2)	−0.33851 (19)	0.23507 (12)	0.0485 (8)	
C5	0.3308 (2)	−0.2584 (2)	0.25647 (12)	0.0478 (8)	
C6	0.1483 (3)	−0.1551 (3)	0.2193 (2)	0.116 (2)	
C7	0.1288 (4)	−0.3003 (4)	0.1069 (2)	0.152 (3)	
C8	0.3394 (6)	−0.4492 (3)	0.1387 (2)	0.134 (3)	
C9	0.4877 (3)	−0.3916 (3)	0.26950 (18)	0.0806 (13)	
C10	0.3661 (3)	−0.2137 (2)	0.31759 (16)	0.0789 (14)	
C11	0.41167 (19)	−0.05235 (17)	0.16787 (11)	0.0392 (7)	
C12	0.35855 (19)	−0.08679 (18)	0.11030 (11)	0.0448 (8)	
C13	0.4059 (2)	−0.1674 (2)	0.08548 (11)	0.0467 (8)	
C14	0.5055 (2)	−0.21405 (19)	0.11782 (11)	0.0448 (8)	
C15	0.55828 (18)	−0.17788 (19)	0.17584 (11)	0.0424 (7)	
C16	0.51260 (18)	−0.09737 (17)	0.20122 (11)	0.0387 (7)	
C17	0.5543 (3)	−0.3003 (2)	0.09141 (14)	0.0656 (11)	
C18	0.5435 (2)	0.22539 (17)	0.08062 (10)	0.0395 (7)	
C19	0.5006 (2)	0.14069 (19)	0.04764 (12)	0.0488 (8)	
C20	0.3856 (2)	0.1250 (2)	0.02495 (13)	0.0585 (10)	
C21	0.3083 (2)	0.1934 (2)	0.03360 (14)	0.0597 (10)	
C22	0.3467 (2)	0.2793 (2)	0.06503 (13)	0.0523 (9)	
C23	0.4607 (2)	0.29397 (18)	0.08782 (12)	0.0472 (8)	
C24	0.68445 (19)	0.24180 (18)	0.18620 (11)	0.0401 (7)	
C25	0.7050 (2)	0.15665 (19)	0.22291 (11)	0.0470 (8)	
C26	0.6928 (2)	0.1559 (2)	0.28160 (12)	0.0570 (9)	
C27	0.6609 (2)	0.2409 (3)	0.30692 (12)	0.0601 (9)	
C28	0.6447 (2)	0.3258 (2)	0.27362 (12)	0.0565 (9)	
C29	0.6569 (2)	0.3257 (2)	0.21496 (12)	0.0478 (8)	
C30	0.7594 (2)	0.15911 (19)	0.09208 (11)	0.0445 (8)	
C31	0.7530 (3)	0.0572 (2)	0.10306 (12)	0.0551 (9)	
C32	0.8262 (3)	−0.0124 (2)	0.08709 (15)	0.0724 (13)	
C33	0.9071 (3)	0.0175 (3)	0.05938 (18)	0.0859 (16)	
C34	0.9148 (3)	0.1154 (3)	0.04638 (18)	0.0844 (16)	
C35	0.8420 (3)	0.1854 (2)	0.06196 (14)	0.0609 (10)	
C36	0.7198 (2)	0.35392 (18)	0.09646 (10)	0.0414 (7)	
C37	0.6609 (2)	0.41396 (19)	0.04952 (12)	0.0521 (9)	
C38	0.7036 (3)	0.5030 (2)	0.03422 (14)	0.0664 (11)	
C39	0.8096 (3)	0.5350 (2)	0.06544 (16)	0.0697 (11)	
C40	0.8697 (3)	0.4785 (2)	0.11235 (16)	0.0646 (11)	
C41	0.8253 (2)	0.3907 (2)	0.12730 (12)	0.0502 (8)	
B1	0.6782 (2)	0.2436 (2)	0.11327 (12)	0.0396 (8)	
H1	0.40710	0.15720	0.15410	0.0640*	
H2	0.48710	0.13980	0.20850	0.0640*	
H6A	0.10370	−0.13660	0.18030	0.1740*	
H6B	0.18710	−0.09780	0.23900	0.1740*	
H6C	0.10010	−0.18140	0.24300	0.1740*	
H7A	0.06640	−0.33590	0.11540	0.2280*	
H7B	0.15350	−0.33340	0.07520	0.2280*	
H7C	0.10580	−0.23390	0.09450	0.2280*	
H8A	0.41880	−0.46240	0.14640	0.2000*	
H8B	0.31070	−0.43320	0.09700	0.2000*	
H8C	0.30130	−0.50690	0.14850	0.2000*	
H9A	0.53480	−0.34610	0.29670	0.1210*	
H9B	0.52860	−0.41770	0.24210	0.1210*	
H9C	0.46560	−0.44510	0.29190	0.1210*	
H10A	0.34900	−0.14400	0.31530	0.1180*	
H10B	0.44580	−0.22280	0.33310	0.1180*	
H10C	0.32640	−0.24540	0.34380	0.1180*	
H12	0.29130	−0.05600	0.08790	0.0530*	
H13	0.36930	−0.19100	0.04670	0.0580*	
H15	0.62520	−0.20890	0.19810	0.0520*	
H16	0.54930	−0.07300	0.23980	0.0480*	
H17A	0.49440	−0.34020	0.06820	0.0980*	
H17B	0.59940	−0.33970	0.12320	0.0980*	
H17C	0.60040	−0.27620	0.06600	0.0980*	
H19	0.55220	0.09190	0.04040	0.0610*	
H20	0.36030	0.06520	0.00340	0.0700*	
H21	0.23000	0.18280	0.01780	0.0730*	
H22	0.29400	0.32720	0.07140	0.0630*	
H23	0.48470	0.35400	0.10910	0.0570*	
H25	0.72810	0.09770	0.20670	0.0580*	
H26	0.70620	0.09680	0.30460	0.0690*	
H27	0.65060	0.24020	0.34680	0.0720*	
H28	0.62510	0.38570	0.29070	0.0700*	
H29	0.64630	0.38680	0.19320	0.0570*	
H31	0.69630	0.03390	0.12180	0.0670*	
H32	0.81760	−0.08090	0.09560	0.0880*	
H33	0.95810	−0.02930	0.04980	0.1060*	
H34	0.97030	0.13710	0.02710	0.1050*	
H35	0.84950	0.25220	0.05170	0.0740*	
H37	0.58870	0.39320	0.02780	0.0620*	
H38	0.66050	0.54150	0.00290	0.0780*	
H39	0.83900	0.59540	0.05380	0.0860*	
H40	0.94190	0.49950	0.13450	0.0760*	
H41	0.86750	0.35340	0.16020	0.0630*	

### Atomic displacement parameters (Å^2^)

**Table:**

	*U*^11^	*U*^22^	*U*^33^	*U*^12^	*U*^13^	*U*^23^
Ru1	0.0329 (1)	0.0389 (1)	0.0379 (1)	−0.0008 (1)	0.0089 (1)	0.0034 (1)
S1	0.0425 (3)	0.0405 (3)	0.0594 (4)	0.0037 (3)	0.0231 (3)	0.0046 (3)
O1	0.0412 (9)	0.0597 (12)	0.0951 (16)	0.0079 (9)	0.0205 (10)	−0.0026 (11)
O2	0.0820 (13)	0.0559 (12)	0.0603 (13)	0.0125 (10)	0.0367 (11)	0.0065 (9)
N1	0.0574 (12)	0.0420 (12)	0.0680 (15)	−0.0022 (10)	0.0243 (11)	0.0044 (11)
C1	0.0398 (13)	0.0571 (18)	0.095 (3)	−0.0028 (12)	0.0271 (15)	0.0244 (16)
C2	0.0550 (16)	0.081 (2)	0.073 (2)	−0.0356 (17)	−0.0092 (15)	0.0294 (18)
C3	0.087 (2)	0.0449 (15)	0.0618 (19)	−0.0228 (15)	0.0308 (16)	−0.0053 (13)
C4	0.0463 (13)	0.0450 (14)	0.0563 (16)	−0.0011 (11)	0.0167 (12)	0.0145 (12)
C5	0.0509 (14)	0.0463 (14)	0.0538 (16)	−0.0089 (11)	0.0277 (12)	0.0026 (12)
C6	0.0615 (19)	0.093 (3)	0.218 (5)	0.027 (2)	0.082 (3)	0.059 (3)
C7	0.111 (3)	0.196 (6)	0.110 (4)	−0.096 (4)	−0.050 (3)	0.060 (4)
C8	0.262 (7)	0.061 (2)	0.104 (4)	−0.052 (3)	0.095 (4)	−0.038 (2)
C9	0.0648 (18)	0.079 (2)	0.101 (3)	0.0155 (17)	0.0260 (18)	0.040 (2)
C10	0.110 (3)	0.075 (2)	0.067 (2)	−0.031 (2)	0.051 (2)	−0.0106 (17)
C11	0.0377 (11)	0.0360 (12)	0.0475 (14)	0.0003 (9)	0.0174 (10)	0.0048 (10)
C12	0.0390 (11)	0.0497 (14)	0.0459 (14)	0.0015 (11)	0.0104 (10)	0.0120 (11)
C13	0.0471 (13)	0.0575 (15)	0.0354 (13)	−0.0010 (12)	0.0097 (10)	0.0018 (11)
C14	0.0440 (12)	0.0496 (14)	0.0447 (14)	0.0022 (11)	0.0182 (10)	−0.0006 (11)
C15	0.0329 (10)	0.0499 (13)	0.0457 (14)	0.0031 (10)	0.0123 (10)	0.0016 (11)
C16	0.0335 (10)	0.0438 (13)	0.0400 (13)	−0.0021 (10)	0.0113 (9)	0.0037 (10)
C17	0.0694 (18)	0.073 (2)	0.0566 (18)	0.0138 (15)	0.0194 (15)	−0.0141 (15)
C18	0.0465 (12)	0.0375 (13)	0.0334 (12)	−0.0011 (10)	0.0077 (10)	0.0046 (9)
C19	0.0549 (14)	0.0449 (14)	0.0452 (15)	0.0000 (12)	0.0093 (11)	−0.0019 (11)
C20	0.0587 (15)	0.0559 (17)	0.0557 (18)	−0.0139 (14)	0.0035 (13)	−0.0080 (13)
C21	0.0483 (14)	0.068 (2)	0.0575 (18)	−0.0106 (14)	0.0025 (13)	0.0068 (14)
C22	0.0490 (13)	0.0517 (15)	0.0555 (17)	0.0079 (12)	0.0114 (12)	0.0108 (13)
C23	0.0514 (13)	0.0414 (13)	0.0467 (14)	0.0003 (11)	0.0078 (11)	0.0044 (11)
C24	0.0362 (11)	0.0464 (13)	0.0369 (13)	−0.0023 (10)	0.0076 (9)	−0.0011 (10)
C25	0.0472 (13)	0.0493 (15)	0.0436 (14)	−0.0048 (11)	0.0092 (11)	0.0044 (11)
C26	0.0524 (14)	0.0715 (19)	0.0458 (15)	−0.0149 (14)	0.0093 (12)	0.0136 (14)
C27	0.0440 (13)	0.100 (2)	0.0401 (15)	−0.0072 (15)	0.0176 (12)	0.0020 (15)
C28	0.0446 (13)	0.084 (2)	0.0426 (15)	0.0038 (14)	0.0136 (11)	−0.0126 (14)
C29	0.0467 (13)	0.0539 (14)	0.0429 (14)	0.0033 (12)	0.0108 (11)	0.0001 (12)
C30	0.0503 (13)	0.0464 (14)	0.0332 (12)	0.0067 (11)	0.0032 (10)	−0.0049 (10)
C31	0.0695 (17)	0.0480 (15)	0.0430 (15)	0.0075 (13)	0.0042 (13)	−0.0039 (12)
C32	0.104 (3)	0.0500 (17)	0.0499 (18)	0.0252 (17)	−0.0072 (17)	−0.0110 (14)
C33	0.095 (3)	0.090 (3)	0.073 (2)	0.038 (2)	0.021 (2)	−0.021 (2)
C34	0.081 (2)	0.098 (3)	0.084 (3)	0.013 (2)	0.039 (2)	−0.021 (2)
C35	0.0679 (17)	0.0652 (18)	0.0538 (17)	0.0046 (14)	0.0227 (14)	−0.0102 (14)
C36	0.0491 (12)	0.0391 (13)	0.0374 (13)	0.0001 (10)	0.0132 (10)	−0.0032 (10)
C37	0.0621 (15)	0.0479 (15)	0.0449 (15)	−0.0038 (13)	0.0102 (12)	0.0036 (12)
C38	0.096 (2)	0.0490 (16)	0.0559 (19)	−0.0016 (16)	0.0220 (17)	0.0105 (14)
C39	0.101 (2)	0.0458 (16)	0.071 (2)	−0.0191 (17)	0.038 (2)	−0.0041 (15)
C40	0.0671 (17)	0.0595 (18)	0.072 (2)	−0.0203 (15)	0.0264 (16)	−0.0159 (16)
C41	0.0513 (13)	0.0521 (15)	0.0476 (15)	−0.0041 (12)	0.0127 (12)	−0.0036 (12)
B1	0.0445 (13)	0.0385 (13)	0.0343 (14)	0.0006 (11)	0.0069 (11)	0.0003 (11)

### Geometric parameters (Å, °)

**Table:**

Ru1—C1	2.192 (3)	C16—H16	0.9500
Ru1—C2	2.177 (4)	C17—H17A	0.9600
Ru1—C3	2.180 (3)	C17—H17B	0.9600
Ru1—C4	2.190 (3)	C17—H17C	0.9600
Ru1—C5	2.196 (3)	C18—C19	1.399 (3)
Ru1—C11	2.193 (2)	C18—C23	1.409 (3)
Ru1—C12	2.211 (2)	C18—B1	1.654 (4)
Ru1—C13	2.224 (2)	C19—C20	1.392 (4)
Ru1—C14	2.240 (3)	C20—C21	1.368 (4)
Ru1—C15	2.219 (2)	C21—C22	1.384 (4)
Ru1—C16	2.211 (2)	C22—C23	1.379 (4)
S1—O1	1.414 (2)	C24—C25	1.409 (4)
S1—O2	1.418 (2)	C24—C29	1.390 (4)
S1—N1	1.601 (2)	C24—B1	1.655 (4)
S1—C11	1.782 (2)	C25—C26	1.389 (4)
N1—H2	0.8600	C26—C27	1.382 (5)
N1—H1	0.8600	C27—C28	1.362 (5)
C1—C5	1.404 (4)	C28—C29	1.388 (4)
C1—C2	1.407 (5)	C30—C31	1.401 (4)
C1—C6	1.513 (5)	C30—C35	1.400 (4)
C2—C3	1.441 (5)	C30—B1	1.658 (4)
C2—C7	1.518 (6)	C31—C32	1.404 (5)
C3—C8	1.495 (5)	C32—C33	1.359 (5)
C3—C4	1.427 (4)	C33—C34	1.360 (6)
C4—C5	1.415 (4)	C34—C35	1.401 (5)
C4—C9	1.493 (5)	C36—C37	1.398 (4)
C5—C10	1.490 (4)	C36—C41	1.403 (4)
C11—C16	1.421 (3)	C36—B1	1.646 (4)
C11—C12	1.401 (3)	C37—C38	1.386 (4)
C12—C13	1.413 (4)	C38—C39	1.389 (5)
C13—C14	1.411 (4)	C39—C40	1.376 (5)
C14—C15	1.417 (3)	C40—C41	1.379 (4)
C14—C17	1.499 (4)	C19—H19	0.9500
C15—C16	1.408 (3)	C20—H20	0.9600
C6—H6A	0.9600	C21—H21	0.9500
C6—H6B	0.9600	C22—H22	0.9500
C6—H6C	0.9600	C23—H23	0.9500
C7—H7A	0.9600	C25—H25	0.9500
C7—H7B	0.9600	C26—H26	0.9500
C7—H7C	0.9600	C27—H27	0.9500
C8—H8A	0.9600	C28—H28	0.9500
C8—H8B	0.9600	C29—H29	0.9500
C8—H8C	0.9600	C31—H31	0.9500
C9—H9A	0.9600	C32—H32	0.9500
C9—H9B	0.9600	C33—H33	0.9500
C9—H9C	0.9600	C34—H34	0.9400
C10—H10A	0.9600	C35—H35	0.9400
C10—H10B	0.9600	C37—H37	0.9400
C10—H10C	0.9600	C38—H38	0.9400
C12—H12	0.9500	C39—H39	0.9500
C13—H13	0.9500	C40—H40	0.9500
C15—H15	0.9500	C41—H41	0.9500
			
S1···H6B	3.0600	C23···H37	2.6800
O1···C6	3.352 (5)	C23···H1	2.5700
O1···C5^i^	3.269 (3)	C24···H23	3.0500
O1···C10^i^	3.408 (4)	C24···H41	2.8800
O1···C4^i^	3.293 (3)	C24···H2	2.9300
O2···C2^i^	3.303 (4)	C25···H31	2.8300
O2···C3^i^	3.369 (4)	C25···H2	2.6100
O1···H6B	2.7900	C25···H15^v^	3.0100
O1···H10C^i^	2.7900	C26···H15^v^	2.8300
O1···H12	2.5800	C26···H2	2.6700
O1···H9C^i^	2.8600	C26···H17B^v^	2.9200
O2···H16	2.6800	C27···H15^v^	2.7300
O2···H6B	2.8200	C27···H2	3.0200
O2···H10A	2.6500	C28···H15^v^	2.7700
O2···H40^ii^	2.8200	C28···H6C^i^	2.9300
N1···C22	3.349 (4)	C29···H23	2.8300
N1···C23	3.254 (3)	C29···H15^v^	2.9500
N1···C25	3.431 (3)	C30···H25	2.8700
N1···H6C^i^	2.7900	C30···H19	2.6800
C1···C3	2.304 (4)	C31···H25	2.5300
C1···C4	2.293 (4)	C31···H19	2.5700
C1···C7	2.610 (6)	C31···H20^iv^	2.9900
C1···C10	2.572 (5)	C32···H20^iv^	2.7800
C1···C11	3.578 (4)	C32···H28^ii^	3.0500
C2···C4	2.305 (5)	C36···H35	2.5000
C2···C5	2.279 (4)	C36···H29	2.6200
C2···C6	2.604 (6)	C36···H23	2.9600
C2···C8	2.617 (7)	C37···H17A^iv^	3.0600
C2···O2^iii^	3.303 (4)	C37···H23	2.9300
C3···C1	2.304 (4)	C40···H26^v^	2.8100
C3···C5	2.295 (4)	C41···H29	2.9400
C3···C7	2.632 (6)	C41···H16^v^	3.1000
C3···C9	2.604 (5)	C41···H35	2.6100
C3···C14	3.536 (4)	H1···C19	2.9400
C3···O2^iii^	3.369 (4)	H1···C18	2.8000
C4···C1	2.293 (4)	H1···C22	2.5900
C4···C2	2.305 (5)	H1···C20	2.9400
C4···C8	2.611 (5)	H1···C21	2.7800
C4···C10	2.583 (4)	H1···C23	2.5700
C4···C15	3.521 (4)	H2···C25	2.6100
C4···O1^iii^	3.293 (3)	H2···C26	2.6700
C5···C2	2.279 (4)	H2···C24	2.9300
C5···C3	2.295 (4)	H2···C27	3.0200
C5···C6	2.592 (5)	H6A···H7C	2.3700
C5···C9	2.590 (5)	H6A···C7	2.8300
C5···C16	3.551 (3)	H6B···O2	2.8200
C5···O1^iii^	3.269 (3)	H6B···C10	2.9300
C6···O1	3.352 (5)	H6B···H10A	2.3900
C10···O1^iii^	3.408 (4)	H6B···S1	3.0600
C11···C1	3.578 (4)	H6B···O1	2.7900
C11···C13	2.431 (3)	H6C···N1^iii^	2.7900
C11···C15	2.438 (3)	H6C···C28^iii^	2.9300
C11···C14	2.831 (3)	H7B···C8	2.8500
C12···C14	2.459 (4)	H7B···H8B	2.3000
C12···S1	2.772 (3)	H7C···H6A	2.3700
C12···C20	3.519 (4)	H7C···C6	2.9800
C12···C15	2.819 (3)	H8A···C9	2.9000
C12···C16	2.452 (3)	H8A···H9B	2.3600
C13···C11	2.431 (3)	H8B···H7B	2.3000
C13···C15	2.432 (3)	H8B···C7	2.9100
C13···C19^iv^	3.526 (4)	H9A···H10B	2.2500
C13···C16	2.820 (3)	H9A···C10	2.8500
C13···C17	2.529 (4)	H9B···C8	2.9100
C14···C16	2.460 (3)	H9B···H8A	2.3600
C14···C12	2.459 (4)	H9C···O1^iii^	2.8600
C14···C11	2.831 (3)	H10A···O2	2.6500
C14···C3	3.536 (4)	H10A···C6	2.8800
C15···C11	2.438 (3)	H10A···H6B	2.3900
C15···C4	3.521 (4)	H10B···C9	2.8100
C15···C13	2.432 (3)	H10B···H41^ii^	2.4700
C15···C17	2.534 (4)	H10B···H9A	2.2500
C15···C28^ii^	3.535 (3)	H10C···O1^iii^	2.7900
C15···C12	2.819 (3)	H12···O1	2.5800
C15···C27^ii^	3.533 (4)	H13···H17A	2.5000
C16···C12	2.452 (3)	H13···C19^iv^	3.0500
C16···C14	2.460 (3)	H15···H17B	2.4300
C16···C13	2.820 (3)	H15···C27^ii^	2.7300
C16···S1	2.762 (2)	H15···C28^ii^	2.7700
C16···C5	3.551 (3)	H15···C29^ii^	2.9500
C19···C31	3.241 (4)	H15···C25^ii^	3.0100
C19···C13^iv^	3.526 (4)	H15···C26^ii^	2.8300
C20···C32^iv^	3.519 (4)	H16···H41^ii^	2.4800
C20···C12	3.519 (4)	H16···O2	2.6800
C22···N1	3.349 (4)	H16···C41^ii^	3.1000
C23···C29	3.324 (4)	H17A···H37^iv^	2.3000
C23···N1	3.254 (3)	H17A···H13	2.5000
C23···C37	3.227 (4)	H17A···C37^iv^	3.0600
C25···N1	3.431 (3)	H17B···H15	2.4300
C25···C31	3.235 (4)	H17B···C26^ii^	2.9200
C27···C15^v^	3.533 (4)	H17C···C21^iv^	2.9900
C28···C15^v^	3.535 (3)	H17C···C20^iv^	2.9500
C29···C23	3.324 (4)	H19···C30	2.6800
C29···C41	3.323 (4)	H19···C31	2.5700
C31···C19	3.241 (4)	H19···H31	2.3700
C31···C25	3.235 (4)	H20···C31^iv^	2.9900
C32···C20^iv^	3.519 (4)	H20···C32^iv^	2.7800
C35···C41	3.175 (4)	H23···C24	3.0500
C37···C23	3.227 (4)	H23···C29	2.8300
C41···C29	3.323 (4)	H23···C36	2.9600
C41···C35	3.175 (4)	H23···C37	2.9300
C6···H10A	2.8800	H23···H29	2.4400
C6···H7C	2.9800	H23···H37	2.5500
C7···H8B	2.9100	H25···C30	2.8700
C7···H6A	2.8300	H25···C31	2.5300
C8···H9B	2.9100	H25···H31	2.0800
C8···H7B	2.8500	H26···C40^ii^	2.8100
C9···H8A	2.9000	H28···C32^v^	3.0500
C9···H10B	2.8100	H28···H32^v^	2.5700
C10···H9A	2.8500	H29···C36	2.6200
C10···H6B	2.9300	H29···C41	2.9400
C11···H16	2.0700	H29···H23	2.4400
C11···H12	2.0500	H31···C19	2.9500
C12···H13	2.0500	H31···C25	2.8300
C13···H12	2.0600	H31···H19	2.3700
C14···H15	2.0600	H31···H25	2.0800
C14···H13	2.0500	H32···H28^ii^	2.5700
C15···H16	2.0600	H33···H34^vii^	2.5900
C16···H15	2.0500	H34···H33^vii^	2.5900
C18···H1	2.8000	H35···C36	2.5000
C18···H37	2.6800	H35···C41	2.6100
C19···H31	2.9500	H37···C18	2.6800
C19···H13^iv^	3.0500	H37···C23	2.6800
C19···H1	2.9400	H37···H23	2.5500
C20···H17C^iv^	2.9500	H37···H17A^iv^	2.3000
C20···H1	2.9400	H38···C22^vi^	2.8600
C21···H17C^iv^	2.9900	H40···O2^v^	2.8200
C21···H1	2.7800	H41···C24	2.8800
C22···H1	2.5900	H41···H10B^v^	2.4700
C22···H38^vi^	2.8600	H41···H16^v^	2.4800
			
C1—Ru1—C2	37.58 (13)	H6A—C6—H6C	110.00
C1—Ru1—C3	63.62 (12)	H6B—C6—H6C	109.00
C1—Ru1—C4	63.12 (10)	C2—C7—H7A	110.00
C1—Ru1—C5	37.32 (11)	C2—C7—H7B	109.00
C1—Ru1—C11	109.38 (9)	C2—C7—H7C	110.00
C1—Ru1—C12	112.77 (10)	H7A—C7—H7B	109.00
C1—Ru1—C13	135.24 (11)	H7A—C7—H7C	110.00
C1—Ru1—C14	165.69 (11)	H7B—C7—H7C	109.00
C1—Ru1—C15	156.52 (11)	C3—C8—H8A	109.00
C1—Ru1—C16	126.74 (10)	C3—C8—H8B	109.00
C2—Ru1—C3	38.61 (14)	C3—C8—H8C	109.00
C2—Ru1—C4	63.70 (12)	H8A—C8—H8B	110.00
C2—Ru1—C5	62.82 (12)	H8A—C8—H8C	109.00
C2—Ru1—C11	132.08 (12)	H8B—C8—H8C	109.00
C2—Ru1—C12	111.04 (12)	C4—C9—H9A	109.00
C2—Ru1—C13	109.92 (11)	C4—C9—H9B	110.00
C2—Ru1—C14	128.25 (12)	C4—C9—H9C	109.00
C2—Ru1—C15	158.89 (12)	H9A—C9—H9B	109.00
C2—Ru1—C16	163.63 (12)	H9A—C9—H9C	109.00
C3—Ru1—C4	38.11 (11)	H9B—C9—H9C	109.00
C3—Ru1—C5	63.28 (11)	C5—C10—H10A	110.00
C3—Ru1—C11	170.65 (11)	C5—C10—H10B	109.00
C3—Ru1—C12	137.82 (10)	C5—C10—H10C	109.00
C3—Ru1—C13	113.16 (11)	H10A—C10—H10B	109.00
C3—Ru1—C14	106.27 (11)	H10A—C10—H10C	109.00
C3—Ru1—C15	121.88 (12)	H10B—C10—H10C	109.00
C3—Ru1—C16	151.35 (11)	Ru1—C12—H12	130.00
C4—Ru1—C5	37.65 (10)	C11—C12—H12	120.00
C4—Ru1—C11	146.09 (9)	C13—C12—H12	120.00
C4—Ru1—C12	174.73 (9)	Ru1—C13—H13	129.00
C4—Ru1—C13	143.29 (10)	C12—C13—H13	119.00
C4—Ru1—C14	116.01 (9)	C14—C13—H13	120.00
C4—Ru1—C15	105.99 (9)	Ru1—C15—H15	129.00
C4—Ru1—C16	117.58 (9)	C14—C15—H15	119.00
C5—Ru1—C11	115.51 (10)	C16—C15—H15	120.00
C5—Ru1—C12	140.79 (9)	Ru1—C16—H16	130.00
C5—Ru1—C13	172.28 (9)	C11—C16—H16	120.00
C5—Ru1—C14	149.41 (9)	C15—C16—H16	120.00
C5—Ru1—C15	121.35 (9)	C14—C17—H17A	109.00
C5—Ru1—C16	107.40 (9)	C14—C17—H17B	109.00
C11—Ru1—C12	37.11 (9)	C14—C17—H17C	109.00
C11—Ru1—C13	66.77 (9)	H17A—C17—H17B	109.00
C11—Ru1—C14	79.37 (9)	H17A—C17—H17C	110.00
C11—Ru1—C15	67.11 (9)	H17B—C17—H17C	109.00
C11—Ru1—C16	37.64 (9)	C19—C18—C23	114.3 (2)
C12—Ru1—C13	37.16 (9)	C19—C18—B1	124.6 (2)
C12—Ru1—C14	67.07 (9)	C23—C18—B1	121.0 (2)
C12—Ru1—C15	79.06 (9)	C18—C19—C20	122.6 (2)
C12—Ru1—C16	67.35 (9)	C19—C20—C21	121.0 (3)
C13—Ru1—C14	36.85 (9)	C20—C21—C22	118.6 (2)
C13—Ru1—C15	66.36 (9)	C21—C22—C23	120.1 (2)
C13—Ru1—C16	78.98 (9)	C18—C23—C22	123.4 (2)
C14—Ru1—C15	37.05 (9)	C25—C24—C29	114.0 (2)
C14—Ru1—C16	67.11 (9)	C25—C24—B1	125.0 (2)
C15—Ru1—C16	37.08 (9)	C29—C24—B1	120.7 (2)
O1—S1—O2	120.63 (14)	C24—C25—C26	122.6 (2)
O1—S1—N1	107.73 (13)	C25—C26—C27	120.5 (3)
O1—S1—C11	106.42 (12)	C26—C27—C28	118.5 (2)
O2—S1—N1	106.94 (13)	C27—C28—C29	120.4 (3)
O2—S1—C11	107.28 (12)	C24—C29—C28	123.7 (2)
N1—S1—C11	107.20 (12)	C31—C30—C35	114.5 (3)
H1—N1—H2	109.00	C31—C30—B1	123.8 (2)
S1—N1—H1	110.00	C35—C30—B1	121.7 (2)
S1—N1—H2	110.00	C30—C31—C32	122.7 (3)
Ru1—C1—C2	70.7 (2)	C31—C32—C33	120.4 (3)
C2—C1—C6	126.1 (3)	C32—C33—C34	119.1 (3)
C5—C1—C6	125.4 (3)	C33—C34—C35	121.0 (3)
Ru1—C1—C6	126.8 (2)	C30—C35—C34	122.3 (3)
C2—C1—C5	108.4 (3)	C37—C36—C41	115.0 (2)
Ru1—C1—C5	71.50 (15)	C37—C36—B1	124.9 (2)
Ru1—C2—C1	71.77 (19)	C41—C36—B1	120.0 (2)
Ru1—C2—C3	70.8 (2)	C36—C37—C38	122.7 (3)
C3—C2—C7	125.6 (3)	C37—C38—C39	120.0 (3)
C1—C2—C7	126.3 (4)	C38—C39—C40	119.1 (3)
Ru1—C2—C7	126.4 (3)	C39—C40—C41	120.1 (3)
C1—C2—C3	108.0 (3)	C36—C41—C40	123.2 (3)
Ru1—C3—C2	70.59 (19)	C20—C19—H19	119.00
Ru1—C3—C4	71.33 (16)	C18—C19—H19	119.00
Ru1—C3—C8	127.6 (3)	C19—C20—H20	119.00
C2—C3—C4	107.0 (3)	C21—C20—H20	120.00
C2—C3—C8	126.1 (4)	C22—C21—H21	121.00
C4—C3—C8	126.6 (3)	C20—C21—H21	121.00
Ru1—C4—C5	71.39 (15)	C21—C22—H22	119.00
C3—C4—C9	126.2 (3)	C23—C22—H22	120.00
Ru1—C4—C9	126.8 (2)	C18—C23—H23	118.00
C3—C4—C5	107.7 (2)	C22—C23—H23	118.00
Ru1—C4—C3	70.57 (15)	C26—C25—H25	119.00
C5—C4—C9	125.9 (3)	C24—C25—H25	118.00
C1—C5—C4	108.9 (2)	C25—C26—H26	120.00
C1—C5—C10	125.5 (3)	C27—C26—H26	119.00
Ru1—C5—C10	127.6 (2)	C26—C27—H27	121.00
Ru1—C5—C1	71.19 (17)	C28—C27—H27	121.00
Ru1—C5—C4	70.96 (15)	C29—C28—H28	120.00
C4—C5—C10	125.5 (2)	C27—C28—H28	120.00
Ru1—C11—S1	126.90 (13)	C24—C29—H29	118.00
Ru1—C11—C12	72.16 (14)	C28—C29—H29	118.00
C12—C11—C16	120.7 (2)	C30—C31—H31	119.00
Ru1—C11—C16	71.86 (13)	C32—C31—H31	118.00
S1—C11—C12	120.63 (18)	C33—C32—H32	121.00
S1—C11—C16	118.70 (18)	C31—C32—H32	119.00
Ru1—C12—C11	70.73 (14)	C32—C33—H33	120.00
Ru1—C12—C13	71.93 (14)	C34—C33—H33	121.00
C11—C12—C13	119.5 (2)	C35—C34—H34	119.00
C12—C13—C14	121.1 (2)	C33—C34—H34	120.00
Ru1—C13—C12	70.92 (14)	C30—C35—H35	119.00
Ru1—C13—C14	72.17 (14)	C34—C35—H35	118.00
Ru1—C14—C15	70.68 (14)	C36—C37—H37	118.00
C13—C14—C17	120.7 (2)	C38—C37—H37	119.00
Ru1—C14—C17	129.90 (19)	C39—C38—H38	120.00
C13—C14—C15	118.6 (2)	C37—C38—H38	120.00
Ru1—C14—C13	70.99 (14)	C38—C39—H39	119.00
C15—C14—C17	120.7 (2)	C40—C39—H39	122.00
C14—C15—C16	121.1 (2)	C39—C40—H40	120.00
Ru1—C15—C14	72.27 (14)	C41—C40—H40	120.00
Ru1—C15—C16	71.14 (13)	C40—C41—H41	119.00
Ru1—C16—C11	70.49 (13)	C36—C41—H41	118.00
Ru1—C16—C15	71.78 (13)	C18—B1—C30	111.52 (19)
C11—C16—C15	119.1 (2)	C18—B1—C36	110.98 (19)
C1—C6—H6A	109.00	C24—B1—C36	107.95 (19)
C1—C6—H6B	109.00	C30—B1—C36	108.07 (19)
C1—C6—H6C	109.00	C24—B1—C30	113.65 (19)
H6A—C6—H6B	110.00	C18—B1—C24	104.62 (18)
			
C2—Ru1—C1—C5	118.0 (3)	C3—Ru1—C14—C15	−121.77 (16)
C2—Ru1—C1—C6	−121.2 (4)	C3—Ru1—C14—C17	−7.5 (3)
C3—Ru1—C1—C2	−38.2 (2)	C4—Ru1—C14—C13	146.76 (15)
C3—Ru1—C1—C5	79.79 (18)	C4—Ru1—C14—C15	−82.09 (16)
C3—Ru1—C1—C6	−159.4 (4)	C4—Ru1—C14—C17	32.2 (3)
C4—Ru1—C1—C2	−81.1 (2)	C5—Ru1—C14—C13	171.82 (18)
C4—Ru1—C1—C5	36.95 (15)	C5—Ru1—C14—C15	−57.0 (2)
C4—Ru1—C1—C6	157.8 (4)	C5—Ru1—C14—C17	57.3 (3)
C5—Ru1—C1—C2	−118.0 (3)	C11—Ru1—C14—C13	−65.24 (15)
C5—Ru1—C1—C6	120.8 (4)	C11—Ru1—C14—C15	65.90 (15)
C11—Ru1—C1—C2	135.1 (2)	C11—Ru1—C14—C17	−179.8 (3)
C11—Ru1—C1—C5	−106.96 (16)	C12—Ru1—C14—C13	−28.54 (15)
C11—Ru1—C1—C6	13.9 (3)	C12—Ru1—C14—C15	102.61 (16)
C12—Ru1—C1—C2	95.3 (2)	C12—Ru1—C14—C17	−143.1 (3)
C12—Ru1—C1—C5	−146.67 (15)	C13—Ru1—C14—C15	131.2 (2)
C12—Ru1—C1—C6	−25.8 (4)	C13—Ru1—C14—C17	−114.6 (3)
C13—Ru1—C1—C2	58.9 (2)	C15—Ru1—C14—C13	−131.2 (2)
C13—Ru1—C1—C5	176.87 (15)	C15—Ru1—C14—C17	114.3 (3)
C13—Ru1—C1—C6	−62.3 (4)	C16—Ru1—C14—C13	−102.56 (16)
C15—Ru1—C1—C2	−148.0 (3)	C16—Ru1—C14—C15	28.59 (14)
C15—Ru1—C1—C5	−30.0 (3)	C16—Ru1—C14—C17	142.9 (3)
C15—Ru1—C1—C6	90.9 (4)	C1—Ru1—C15—C14	170.8 (2)
C16—Ru1—C1—C2	173.34 (19)	C1—Ru1—C15—C16	−56.2 (3)
C16—Ru1—C1—C5	−68.67 (18)	C2—Ru1—C15—C14	54.7 (4)
C16—Ru1—C1—C6	52.2 (4)	C2—Ru1—C15—C16	−172.3 (3)
C1—Ru1—C2—C3	−117.4 (3)	C3—Ru1—C15—C14	73.96 (18)
C1—Ru1—C2—C7	122.1 (5)	C3—Ru1—C15—C16	−153.02 (15)
C3—Ru1—C2—C1	117.4 (3)	C4—Ru1—C15—C14	112.18 (15)
C3—Ru1—C2—C7	−120.6 (4)	C4—Ru1—C15—C16	−114.81 (15)
C4—Ru1—C2—C1	79.4 (2)	C5—Ru1—C15—C14	150.01 (15)
C4—Ru1—C2—C3	−38.03 (18)	C5—Ru1—C15—C16	−76.98 (17)
C4—Ru1—C2—C7	−158.6 (4)	C11—Ru1—C15—C14	−103.12 (16)
C5—Ru1—C2—C1	36.99 (18)	C11—Ru1—C15—C16	29.90 (14)
C5—Ru1—C2—C3	−80.38 (19)	C12—Ru1—C15—C14	−66.26 (15)
C5—Ru1—C2—C7	159.0 (4)	C12—Ru1—C15—C16	66.76 (15)
C11—Ru1—C2—C1	−63.9 (2)	C13—Ru1—C15—C14	−29.53 (15)
C11—Ru1—C2—C3	178.74 (16)	C13—Ru1—C15—C16	103.48 (16)
C11—Ru1—C2—C7	58.2 (4)	C14—Ru1—C15—C16	133.0 (2)
C12—Ru1—C2—C1	−100.4 (2)	C16—Ru1—C15—C14	−133.0 (2)
C12—Ru1—C2—C3	142.25 (17)	C1—Ru1—C16—C11	−73.15 (18)
C12—Ru1—C2—C7	21.7 (4)	C1—Ru1—C16—C15	155.60 (16)
C13—Ru1—C2—C1	−140.12 (18)	C3—Ru1—C16—C11	−175.3 (2)
C13—Ru1—C2—C3	102.50 (19)	C3—Ru1—C16—C15	53.5 (3)
C13—Ru1—C2—C7	−18.1 (4)	C4—Ru1—C16—C11	−148.87 (14)
C14—Ru1—C2—C1	−177.17 (17)	C4—Ru1—C16—C15	79.88 (16)
C14—Ru1—C2—C3	65.5 (2)	C5—Ru1—C16—C11	−109.44 (15)
C14—Ru1—C2—C7	−55.1 (4)	C5—Ru1—C16—C15	119.31 (15)
C15—Ru1—C2—C1	144.1 (3)	C11—Ru1—C16—C15	−131.3 (2)
C15—Ru1—C2—C3	26.7 (4)	C12—Ru1—C16—C11	29.07 (14)
C15—Ru1—C2—C7	−93.9 (5)	C12—Ru1—C16—C15	−102.18 (16)
C1—Ru1—C3—C2	37.2 (2)	C13—Ru1—C16—C11	66.08 (15)
C1—Ru1—C3—C4	−79.31 (19)	C13—Ru1—C16—C15	−65.17 (15)
C1—Ru1—C3—C8	158.4 (4)	C14—Ru1—C16—C11	102.69 (15)
C2—Ru1—C3—C4	−116.5 (3)	C14—Ru1—C16—C15	−28.57 (14)
C2—Ru1—C3—C8	121.2 (4)	C15—Ru1—C16—C11	131.3 (2)
C4—Ru1—C3—C2	116.5 (3)	O1—S1—C11—Ru1	−73.93 (18)
C4—Ru1—C3—C8	−122.3 (4)	O1—S1—C11—C12	16.0 (2)
C5—Ru1—C3—C2	79.1 (2)	O1—S1—C11—C16	−162.05 (19)
C5—Ru1—C3—C4	−37.40 (16)	O2—S1—C11—Ru1	56.45 (18)
C5—Ru1—C3—C8	−159.7 (4)	O2—S1—C11—C12	146.4 (2)
C12—Ru1—C3—C2	−58.3 (3)	O2—S1—C11—C16	−31.7 (2)
C12—Ru1—C3—C4	−174.81 (14)	N1—S1—C11—Ru1	171.02 (15)
C12—Ru1—C3—C8	62.9 (4)	N1—S1—C11—C12	−99.0 (2)
C13—Ru1—C3—C2	−93.3 (2)	N1—S1—C11—C16	82.9 (2)
C13—Ru1—C3—C4	150.16 (16)	Ru1—C1—C2—C3	61.9 (2)
C13—Ru1—C3—C8	27.8 (4)	Ru1—C1—C2—C7	−122.3 (4)
C14—Ru1—C3—C2	−131.91 (19)	C5—C1—C2—Ru1	−61.9 (2)
C14—Ru1—C3—C4	111.59 (17)	C5—C1—C2—C3	0.0 (4)
C14—Ru1—C3—C8	−10.7 (4)	C5—C1—C2—C7	175.9 (4)
C15—Ru1—C3—C2	−169.01 (18)	C6—C1—C2—Ru1	122.0 (3)
C15—Ru1—C3—C4	74.49 (19)	C6—C1—C2—C3	−176.2 (3)
C15—Ru1—C3—C8	−47.8 (4)	C6—C1—C2—C7	−0.3 (6)
C16—Ru1—C3—C2	156.2 (2)	Ru1—C1—C5—C4	−61.27 (19)
C16—Ru1—C3—C4	39.7 (3)	Ru1—C1—C5—C10	123.3 (3)
C16—Ru1—C3—C8	−82.6 (4)	C2—C1—C5—Ru1	61.4 (2)
C1—Ru1—C4—C3	80.8 (2)	C2—C1—C5—C4	0.1 (3)
C1—Ru1—C4—C5	−36.63 (16)	C2—C1—C5—C10	−175.3 (3)
C1—Ru1—C4—C9	−158.0 (3)	C6—C1—C5—Ru1	−122.5 (3)
C2—Ru1—C4—C3	38.5 (2)	C6—C1—C5—C4	176.3 (3)
C2—Ru1—C4—C5	−78.85 (18)	C6—C1—C5—C10	0.8 (5)
C2—Ru1—C4—C9	159.8 (3)	Ru1—C2—C3—C4	62.4 (2)
C3—Ru1—C4—C5	−117.4 (2)	Ru1—C2—C3—C8	−122.9 (4)
C3—Ru1—C4—C9	121.2 (3)	C1—C2—C3—Ru1	−62.5 (2)
C5—Ru1—C4—C3	117.4 (2)	C1—C2—C3—C4	−0.1 (4)
C5—Ru1—C4—C9	−121.4 (3)	C1—C2—C3—C8	174.6 (4)
C11—Ru1—C4—C3	165.74 (19)	C7—C2—C3—Ru1	121.6 (4)
C11—Ru1—C4—C5	48.4 (2)	C7—C2—C3—C4	−176.0 (4)
C11—Ru1—C4—C9	−73.0 (3)	C7—C2—C3—C8	−1.3 (6)
C13—Ru1—C4—C3	−49.9 (2)	Ru1—C3—C4—C5	62.08 (19)
C13—Ru1—C4—C5	−167.30 (16)	Ru1—C3—C4—C9	−121.9 (3)
C13—Ru1—C4—C9	71.3 (3)	C2—C3—C4—Ru1	−62.0 (2)
C14—Ru1—C4—C3	−83.29 (19)	C2—C3—C4—C5	0.1 (3)
C14—Ru1—C4—C5	159.34 (14)	C2—C3—C4—C9	176.1 (3)
C14—Ru1—C4—C9	37.9 (3)	C8—C3—C4—Ru1	123.4 (4)
C15—Ru1—C4—C3	−121.66 (18)	C8—C3—C4—C5	−174.5 (4)
C15—Ru1—C4—C5	120.96 (15)	C8—C3—C4—C9	1.5 (6)
C15—Ru1—C4—C9	−0.4 (3)	Ru1—C4—C5—C1	61.41 (19)
C16—Ru1—C4—C3	−159.79 (18)	Ru1—C4—C5—C10	−123.2 (3)
C16—Ru1—C4—C5	82.84 (16)	C3—C4—C5—Ru1	−61.6 (2)
C16—Ru1—C4—C9	−38.6 (3)	C3—C4—C5—C1	−0.1 (3)
C1—Ru1—C5—C4	118.6 (2)	C3—C4—C5—C10	175.3 (3)
C1—Ru1—C5—C10	−120.7 (3)	C9—C4—C5—Ru1	122.4 (3)
C2—Ru1—C5—C1	−37.25 (18)	C9—C4—C5—C1	−176.2 (3)
C2—Ru1—C5—C4	81.38 (19)	C9—C4—C5—C10	−0.7 (5)
C2—Ru1—C5—C10	−158.0 (3)	Ru1—C11—C12—C13	−54.9 (2)
C3—Ru1—C5—C1	−80.79 (19)	S1—C11—C12—Ru1	−122.85 (18)
C3—Ru1—C5—C4	37.85 (17)	S1—C11—C12—C13	−177.76 (19)
C3—Ru1—C5—C10	158.5 (3)	C16—C11—C12—Ru1	55.2 (2)
C4—Ru1—C5—C1	−118.6 (2)	C16—C11—C12—C13	0.3 (4)
C4—Ru1—C5—C10	120.6 (3)	Ru1—C11—C16—C15	54.8 (2)
C11—Ru1—C5—C1	88.88 (17)	S1—C11—C16—Ru1	122.75 (17)
C11—Ru1—C5—C4	−152.49 (14)	S1—C11—C16—C15	177.54 (18)
C11—Ru1—C5—C10	−31.9 (3)	C12—C11—C16—Ru1	−55.3 (2)
C12—Ru1—C5—C1	53.3 (2)	C12—C11—C16—C15	−0.5 (3)
C12—Ru1—C5—C4	171.90 (15)	Ru1—C12—C13—C14	−54.1 (2)
C12—Ru1—C5—C10	−67.5 (3)	C11—C12—C13—Ru1	54.3 (2)
C14—Ru1—C5—C1	−157.18 (18)	C11—C12—C13—C14	0.3 (4)
C14—Ru1—C5—C4	−38.6 (3)	Ru1—C13—C14—C15	−54.1 (2)
C14—Ru1—C5—C10	82.1 (3)	Ru1—C13—C14—C17	125.8 (2)
C15—Ru1—C5—C1	166.53 (15)	C12—C13—C14—Ru1	53.5 (2)
C15—Ru1—C5—C4	−74.84 (17)	C12—C13—C14—C15	−0.6 (4)
C15—Ru1—C5—C10	45.8 (3)	C12—C13—C14—C17	179.3 (2)
C16—Ru1—C5—C1	128.54 (16)	Ru1—C14—C15—C16	−53.9 (2)
C16—Ru1—C5—C4	−112.83 (15)	C13—C14—C15—Ru1	54.2 (2)
C16—Ru1—C5—C10	7.8 (3)	C13—C14—C15—C16	0.3 (4)
C1—Ru1—C11—S1	12.9 (2)	C17—C14—C15—Ru1	−125.6 (2)
C1—Ru1—C11—C12	−102.41 (17)	C17—C14—C15—C16	−179.5 (2)
C1—Ru1—C11—C16	125.61 (16)	Ru1—C15—C16—C11	−54.2 (2)
C2—Ru1—C11—S1	48.4 (2)	C14—C15—C16—Ru1	54.4 (2)
C2—Ru1—C11—C12	−66.9 (2)	C14—C15—C16—C11	0.2 (4)
C2—Ru1—C11—C16	161.09 (17)	C23—C18—C19—C20	−1.2 (4)
C4—Ru1—C11—S1	−57.5 (2)	B1—C18—C19—C20	174.8 (2)
C4—Ru1—C11—C12	−172.78 (16)	C19—C18—C23—C22	0.6 (4)
C4—Ru1—C11—C16	55.2 (2)	B1—C18—C23—C22	−175.5 (2)
C5—Ru1—C11—S1	−27.07 (19)	C19—C18—B1—C24	−112.9 (3)
C5—Ru1—C11—C12	−142.39 (14)	C19—C18—B1—C30	10.3 (3)
C5—Ru1—C11—C16	85.63 (15)	C19—C18—B1—C36	130.9 (2)
C12—Ru1—C11—S1	115.3 (2)	C23—C18—B1—C24	62.8 (3)
C12—Ru1—C11—C16	−132.0 (2)	C23—C18—B1—C30	−174.0 (2)
C13—Ru1—C11—S1	144.83 (18)	C23—C18—B1—C36	−53.4 (3)
C13—Ru1—C11—C12	29.51 (14)	C18—C19—C20—C21	0.6 (4)
C13—Ru1—C11—C16	−102.47 (15)	C19—C20—C21—C22	0.5 (4)
C14—Ru1—C11—S1	−178.83 (17)	C20—C21—C22—C23	−1.1 (4)
C14—Ru1—C11—C12	65.85 (15)	C21—C22—C23—C18	0.5 (4)
C14—Ru1—C11—C16	−66.13 (14)	C29—C24—C25—C26	3.2 (4)
C15—Ru1—C11—S1	−142.17 (18)	B1—C24—C25—C26	−170.1 (2)
C15—Ru1—C11—C12	102.51 (15)	C25—C24—C29—C28	−3.1 (4)
C15—Ru1—C11—C16	−29.47 (14)	B1—C24—C29—C28	170.5 (2)
C16—Ru1—C11—S1	−112.7 (2)	C25—C24—B1—C18	91.4 (3)
C16—Ru1—C11—C12	132.0 (2)	C25—C24—B1—C30	−30.5 (3)
C1—Ru1—C12—C11	92.39 (16)	C25—C24—B1—C36	−150.4 (2)
C1—Ru1—C12—C13	−136.15 (16)	C29—C24—B1—C18	−81.5 (3)
C2—Ru1—C12—C11	132.98 (16)	C29—C24—B1—C30	156.6 (2)
C2—Ru1—C12—C13	−95.57 (17)	C29—C24—B1—C36	36.7 (3)
C3—Ru1—C12—C11	167.65 (18)	C24—C25—C26—C27	−0.9 (4)
C3—Ru1—C12—C13	−60.9 (2)	C25—C26—C27—C28	−1.7 (4)
C5—Ru1—C12—C11	60.6 (2)	C26—C27—C28—C29	1.9 (4)
C5—Ru1—C12—C13	−167.95 (15)	C27—C28—C29—C24	0.7 (4)
C11—Ru1—C12—C13	131.5 (2)	C35—C30—C31—C32	2.2 (4)
C13—Ru1—C12—C11	−131.5 (2)	B1—C30—C31—C32	−177.0 (3)
C14—Ru1—C12—C11	−103.14 (16)	C31—C30—C35—C34	−2.4 (4)
C14—Ru1—C12—C13	28.31 (14)	B1—C30—C35—C34	176.9 (3)
C15—Ru1—C12—C11	−66.35 (15)	C31—C30—B1—C18	−62.2 (3)
C15—Ru1—C12—C13	65.10 (15)	C31—C30—B1—C24	55.8 (3)
C16—Ru1—C12—C11	−29.47 (14)	C31—C30—B1—C36	175.6 (2)
C16—Ru1—C12—C13	101.99 (16)	C35—C30—B1—C18	118.7 (3)
C1—Ru1—C13—C12	65.1 (2)	C35—C30—B1—C24	−123.4 (3)
C1—Ru1—C13—C14	−161.64 (15)	C35—C30—B1—C36	−3.6 (3)
C2—Ru1—C13—C12	98.85 (18)	C30—C31—C32—C33	−0.5 (5)
C2—Ru1—C13—C14	−127.90 (17)	C31—C32—C33—C34	−1.2 (6)
C3—Ru1—C13—C12	140.35 (16)	C32—C33—C34—C35	1.0 (6)
C3—Ru1—C13—C14	−86.41 (18)	C33—C34—C35—C30	0.9 (6)
C4—Ru1—C13—C12	171.26 (15)	C41—C36—C37—C38	0.4 (4)
C4—Ru1—C13—C14	−55.5 (2)	B1—C36—C37—C38	−174.9 (3)
C11—Ru1—C13—C12	−29.47 (14)	C37—C36—C41—C40	−1.1 (4)
C11—Ru1—C13—C14	103.77 (16)	B1—C36—C41—C40	174.4 (3)
C12—Ru1—C13—C14	133.3 (2)	C37—C36—B1—C18	−15.6 (3)
C14—Ru1—C13—C12	−133.3 (2)	C37—C36—B1—C24	−129.7 (2)
C15—Ru1—C13—C12	−103.56 (16)	C37—C36—B1—C30	107.0 (3)
C15—Ru1—C13—C14	29.69 (15)	C41—C36—B1—C18	169.4 (2)
C16—Ru1—C13—C12	−66.88 (15)	C41—C36—B1—C24	55.2 (3)
C16—Ru1—C13—C14	66.36 (15)	C41—C36—B1—C30	−68.1 (3)
C2—Ru1—C14—C13	70.8 (2)	C36—C37—C38—C39	1.0 (5)
C2—Ru1—C14—C15	−158.02 (17)	C37—C38—C39—C40	−1.6 (5)
C2—Ru1—C14—C17	−43.7 (3)	C38—C39—C40—C41	0.9 (5)
C3—Ru1—C14—C13	107.08 (17)	C39—C40—C41—C36	0.5 (5)

Symmetry codes: (i) −*x*+1/2, *y*+1/2, −*z*+1/2; (ii) −*x*+3/2, *y*−1/2, −*z*+1/2; (iii) −*x*+1/2, *y*−1/2, −*z*+1/2; (iv) −*x*+1, −*y*, −*z*; (v) −*x*+3/2, *y*+1/2, −*z*+1/2; (vi) −*x*+1, −*y*+1, −*z*; (vii) −*x*+2, −*y*, −*z*.
